# Prenatal diagnosis of tibial hemimelia type I and omphalocele, a rare entity and postnatal correlation

**DOI:** 10.1259/bjrcr.20200002

**Published:** 2020-09-04

**Authors:** Saulo Molina-Giraldo, Jesus Armando Saucedo, Antonio José Navarro-Devia, Marcela Buitrago-Leal

**Affiliations:** 1 Department of Obstetrics and Gynecology, Division of Maternal Fetal Medicine, Section of Fetal Therapy and Fetal Surgery Unit, Clinica de la Mujer, Bogota, Colombia; 2 Fundacion Universitaria de Ciencias de la Salud, FUCS, Bogota, Colombia; 3 Tecnologico de Monterrey, Escuela de Medicina y CIencias de la Salud, Monterrey, Mexico

## Abstract

Hemimelia is a rare anomaly affecting the distal long bones of extremities, with an occurrence of 1–20 cases per million of live births depending on the affected bone. Hemimelia can be an isolated defect or be part of complex syndromes that affect extra skeletal structures. Prenatal detection by routine ultrasound imaging is difficult and yields low detection rates. The prenatal diagnosis of hemimelia should prompt a complete and detailed study of the fetal anatomy, since it can be associated with defects in other structures and systems, as the reported in this case. The prognosis depends upon the associated anomalies.

## Case report

**Figure 1.**
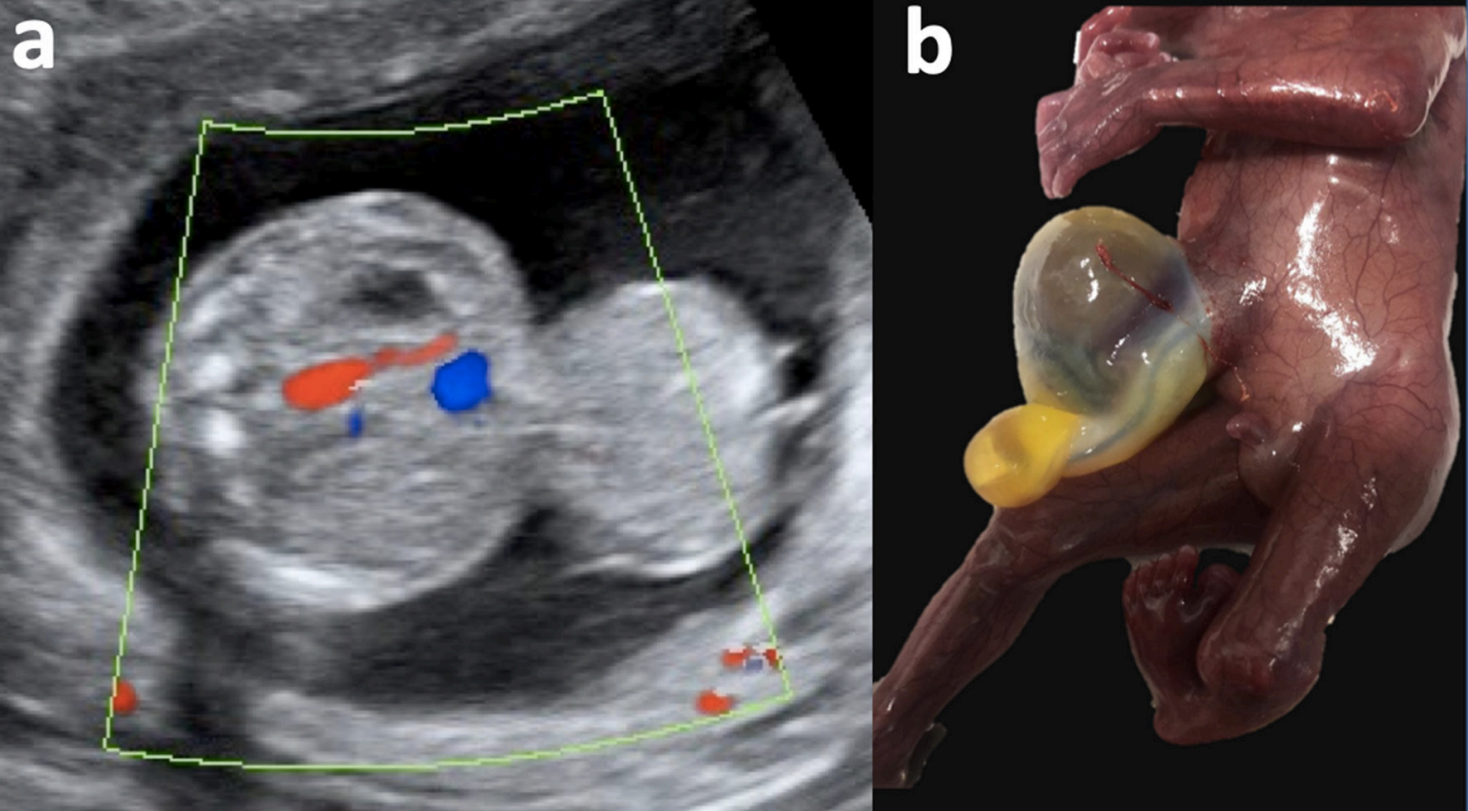
a: 2D Gray scale 2D ultrasound axial abdominal plane demonstrating omphalocele containing hepatic and intestinal content. Figure 1-b: Omphalocele on macroscopic examination of fetal specimen. 2D, two-dimensional.

**Figure 2.**
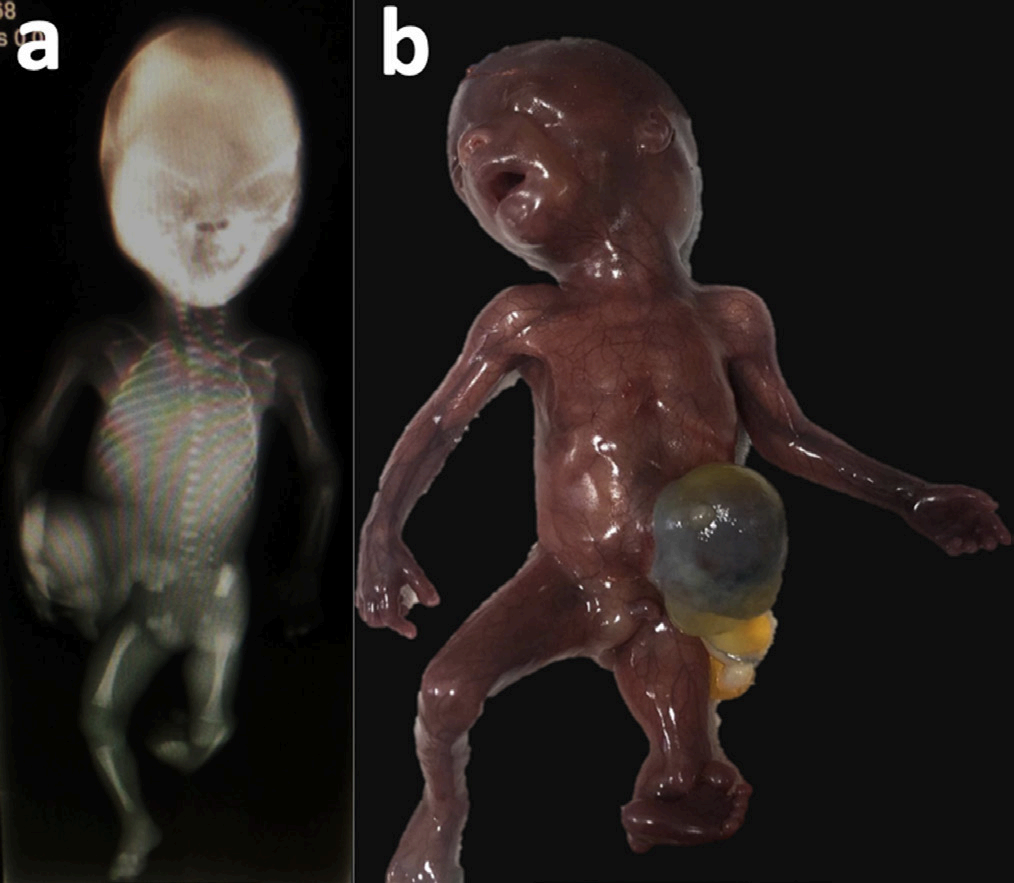
a: Complete body X-ray showing tibial hemimelia present in left extremity. Right lower limb shows the presence of the femur, fibula is not shown due to projection phenomena. Figure 2-b: Fetal specimen, omphalocele, shortening of the left limb and clubfoot can be noted.

**Figure 3.**
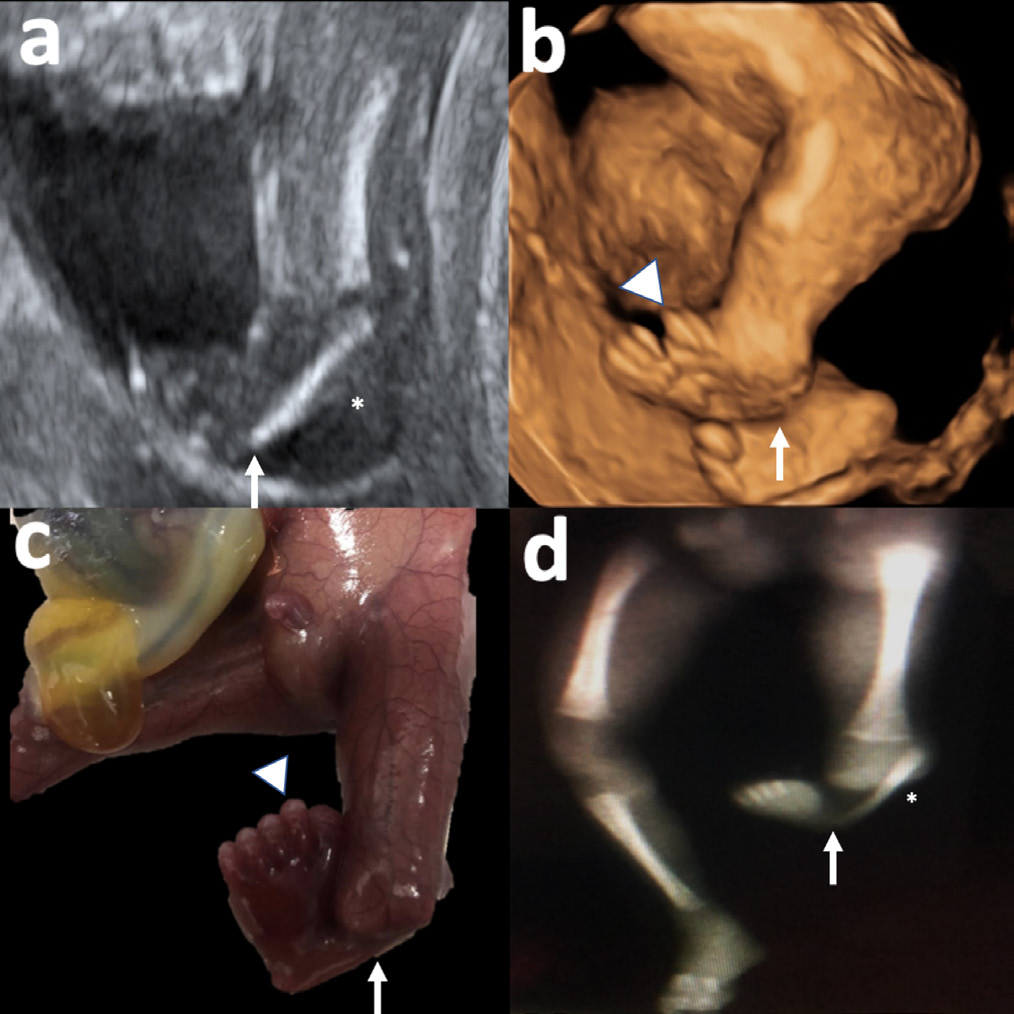
Tibial hemimelia, short fibula (*), clubfoot (arrow), complete pre-axial polydactyly (arrowhead). 3-a: 2D gray scale ultrasound. 3-b: Three-dimensional reconstruction. 3 c: Fetal specimen. 3-d: Postnatal X-ray. Right lower limb displays only tibia because of projection phenomena, fibula is present and of normal characteristics. 2D, two-dimensional.

**Figure 4.**
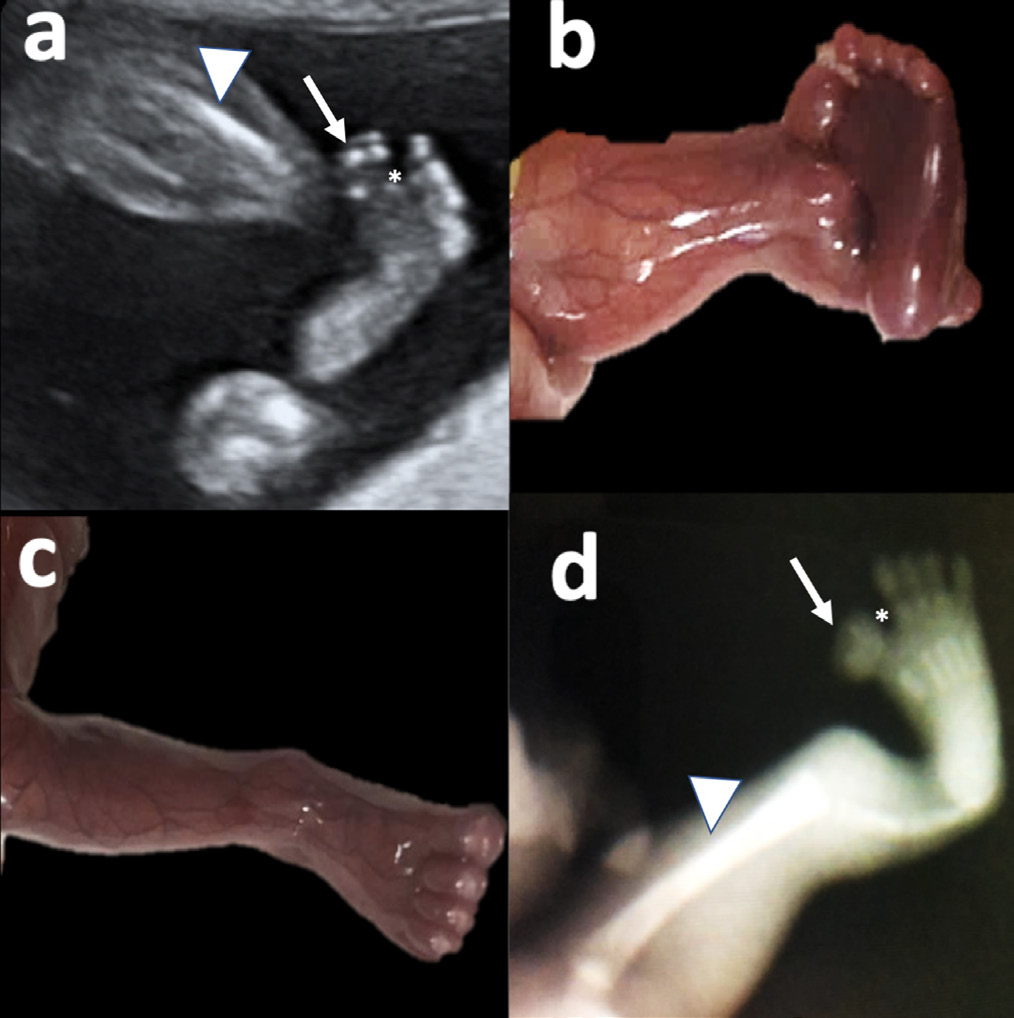
a: Coronal plane of the left foot in 2D Ultrasound. Complete pre-axial polydactyly (arrow), sandal-gap like sign (*), short fibula (arrowhead) and tibial hemimelia are observed. Figure 4 b: Fetal specimen, showing left lower limb with pre-axial polydactyly and sandal-gap sign. Figure 4 c: Fetal specimen, syndactyly in right foot is depicted.Figure 4: X-ray confirming 6 fingers and five metatarsals on the left foot. 2D, two-dimensional.

30-year-old female, gravida 2, with previous cesarean section delivering a healthy newborn at term. She had no known history of malformations, no exposure to drugs, teratogenics or ionizing radiation. The patient underwent a routine ultrasonographic evaluation at 14 weeks of gestation and was referred after omphalocele was detected. We evaluated the patient at 17 weeks using standard two-dimensional ultrasound. A single viable fetus with growth restriction was found (estimated fetal weight in <3rd percentile). Initial evaluation found normal central nervous system anatomy, face and profile. Echocardiographic assessment showed a hyperechogenic cardiac focus located in the left ventricle and an atrioventricular septal defect was suspected. Abdominal wall evaluation confirmed omphalocele with liver and intestinal content and a single umbilical artery (Figure 1). Evaluation of the upper limbs was normal. Right lower limb had normal long bones but syndactyly was observed. Abnormalities on the left lower limb included: normal femur, complete agenesis of the tibia (Figure 2), hypoplasia of the fibula with camptomelic changes (<5th percentile), knee flexion contracture and ankle luxation, rigid clubfoot and supination deformity (Figure 3), and complete pre-axial polydactyly and sandal-gap like sign (Figure 4). Amniotic fluid was present in a normal amount. Karyotype in amniotic fluid was normal (46XY). The family opted for termination of pregnancy, in accordance with local laws. Post-mortem pathologic and radiologic evaluation confirmed the abnormalities diagnosed by ultrasound.

## Discussion

Tibial hemimelia (TH), a longitudinal congenital bone deficiency, is a rare anomaly with an estimated occurrence of one case per million live births. It can be total or partial, unilateral or bilateral, presented as an isolated defect or as a manifestation of complex syndromes, with other skeletal and extra skeletal associated alterations.^1^

The term was initially described by Billroth in 1861 and then modified by Dankmeijer in 1935.^2^

Although the etiology is unclear, a consensus exists stating that the critical period for limb development takes place around 4–8 weeks of gestation. However, the complete understanding of the physiopathology has not yet been achieved.^3^

The fibula is always present and can be normal, dysplasic or even duplicated; quadriceps may be normal, with a distal deficiency or its absence; with the same situation applying to the patella and knee ligaments. The knee can be present in complete extension, flexed contraction, or dislocated, while the ankle can be normal or presented as talipes equinovarus (clubfoot).^4^ In 75% of cases, TH is present with ectrodactyly, pre-axial polydactyly, and ulnar aplasia.^5^

Different classification systems of TH have been described. For example, the classification described by Jones in 1978 is based upon the radiological findings and divides TH in to four basic types: Type I - total absence of the tibia, Type II - distal deficiency of the tibia, Type III -proximal deficiency of the tibia, and Type IV - tibial shortening. Other classification systems have been described and are based on the morphology, the associated malformations, and the presence or absence of articular cartilage.^4^

TH has been described as associated to different genetic syndromes such as: Weber syndrome, Langer-Giedion syndrome, tricho-rhino-phalangeal syndrome, tibial hemimelia-diplopodia syndrome, tibial hemimelia and split hand and foot syndrome, and tibial hemimelia micromelia trigonal brachycephaly syndrome. Autosomal dominant and recessive transmission have also been noted.^2^

Different mutations have been identified, for instance, alterations in the gene regulator Sonic-Hedgehog, which has been associated to TH and polydactyly, as in the presented case.^6^

Bilateral TH has been studied as an autosomal dominant syndrome, also including polysyndactyly, clubfeet, triphalangic fingers and radial deviation of the hands; accounting for approximately 25% of the cases of TH. Familiar cases have been reported.^6^

Unilateral TH accounts for 75% of the TH cases. It presents as a sporadic anomaly, although an autosomal recessive transmission with variable degrees of penetrance has been deliberated.^7^

Pre-axial polydactyly of the foot is genetically heterogeneous. In 80–90% of cases, it is presented as a unilateral affection, and in 20% of cases it is associated with other abnormalities.^8^

The association of TH with malformations in extra skeletal systems has been described. No reported cases of TH and omphalocele have been reported on the OMIM database or PubMed to date. OMIM search using tibial hemimelia as principal term reports only 10 genetic syndromes, none of which fully describe the presented case being the Tibia hypoplasia or aplasia with polydactyly as the most concordant syndrome, but it does not include omphalocele. Another possibility, although uncommon but displaying similar features and cannot be discarded, is Werner´s mesomelic dysplasia accompanied with Hirschsprung´s disease or with ventricular septal defect.^9^

Traditionally, the diagnosis of hemimelia is made in the postnatal life through physical exam of the newborn supported by standard X-ray study of the limbs.

The first report of prenatal diagnosis of TH dates back to 1996.^7^

The prenatal detection rates for fibular hemimelia and congenital femoral deficiency (the most common types of hemimelia^10^) range from 4 to 36%, according to different studies^11^ (using two-dimensional and three-dimensional ultrasound examination). No available data about the detection rates for TH were found.

Treatment of the tibial defect is surgical. Techniques vary depending of the type and extensiveness of the lesion and the associated malformations, from disarticulation, Syme amputation, and bone fusions, with total amputation being the last source of treatment.^12^

Prognosis depends on the associated anomalies.

Even though TH is a rare entity, prenatal diagnosis is feasible. Clinicians should be aware of the existence of TH and the incomplete understanding of its physiopathology and etiology. Its detection should warrant a complete and detailed study of the fetal anatomy by a maternal–fetal specialist where available; as TH is not a fatal condition but the associated malformations may alter the prognosis.

## Learning points

- TH is a rare entity, with low detection rates, but a detailed evaluation of fetal limbs should allow its proper diagnosis.
- An integral diagnostic approach is suggested, including a topographic diagnosis (to identify the anatomic region or system affected), syndromatic diagnosis, and finally an etiologic diagnosis should always being taken into account – whether or not a structure is missing, an extra structure is identified, a structure has an abnormal function, and amniotic fluid and growth are normal or altered.
- Although not very common, fetal limb anomalies may be associated with abdominal wall anomalies and cardiac defects.
- The fetal prognosis is variable in cases of tibial hemimelia and greatly depends on the associated anomalies.
